# Travel-related *Salmonella* Agama, Gabon

**DOI:** 10.3201/eid1305.061275

**Published:** 2007-05

**Authors:** Sabine Bélard, Manfred Kist, Michael Ramharter

**Affiliations:** * Albert Schweitzer Hospital, Lambaréné, Gabon; † University of Tübingen, Tübingen, Germany; ‡ University of Freiburg, Freiburg, Germany; § Medical University of Vienna, Vienna, Austria

**Keywords:** Traveler’s diarrhea, Salmonella enterica serovar Agama, salmonellosis, Germany, Gabon, letter

**To the Editor:** Traveler’s diarrhea affects >50% of travelers to regions such as sub-Saharan Africa (*1*). Worldwide, enterotoxigenic *Escherichia coli* is the leading bacterial pathogen that causes traveler’s diarrhea, followed by *Campylobacter jejuni* and then *Salmonella* spp., which are the causative pathogens for ≥25% of traveler’s diarrhea in Africa (*1*). Nontyphoidal salmonellosis is mostly caused by the *Salmonella* serotypes Enteritidis and Typhimurium (*2*). To our knowledge, only a few cases of salmonellosis due to *S.* Agama have been reported in medical literature, none as a travel-related disease (*3*,*4*).

*S.* Agama was characterized in 1956 as a new serotype of *Salmonella enterica* from the feces of the agama lizard (*Agama agama*) in Nigeria (*5*). Subsequently, *S.* Agama was isolated from geckos and mammals in Africa (*4*,*6*,*7*) and the United Kindgom (*8*,*9*). Human infections with *S.* Agama were once reported in Nigeria and related to the lizards as possible reservoirs (*4*). Another clinical case of *S.* Agama infection was described in France in a 9-month-old child with fever and diarrhea (*3*); fruits imported from Africa were discussed as potential source of infection. We report what is, to our knowledge, the first travel-related case of salmonellosis due to *Salmonella* Agama experienced by a tourist who had traveled to Gabon in central Africa.

A previously healthy 25-year-old man in Germany sought treatment for 2 episodes of intermittent fever <39°C, as well as headache, nausea, abdominal pain, diarrhea, arthralgia, and cough. Symptoms started the day he returned from a 1-month trip to Gabon, a country in central Africa, where he stayed with a friend who lives near the Albert Schweitzer Hospital in Lambaréné and took occasional excursions to other areas.

Before traveling, the patient had been immunized against hepatitis A, hepatitis B, yellow fever, polio, typhoid fever, tetanus, measles, and mumps; he reported taking atovaquone-proguanil for malaria prophylaxis during his first 3 weeks in Gabon. While in Gabon, he frequently drank tap water, ate food sold by street vendors, and had repeated fresh water contact while swimming in the Ogooué River. He exhibited no symptoms during his trip.

His first examination was performed 2 weeks after his return to Germany and the onset of symptoms. Physical examination showed no pathologic findings, malaria was excluded by repeated thick blood smears, and in the absence of abnormal laboratory findings a common cold disease was assumed on clinical grounds. No specific treatment was prescribed, and the patient recovered from symptoms except for intermittent mild diarrhea.

Four weeks after his return to Germany, a second episode with reappearance of all former symptoms led to a new examination. At this time, the patient was afebrile, and physical examination showed no pathologic findings. Laboratory values were within the normal range except for C-reactive protein, which was elevated at 47mg/dL (normal value <5 mg/dL). Pneumonia was excluded by radiography, and a stool sample was obtained for parasitologic examination and bacterial culture. The patient was treated with clarithromycin, 500 mg orally twice a day for 7 days, for a presumed upper respiratory tract infection. The patient’s symptoms disappeared.

Stool sample test results were negative for intestinal helminths and other parasites. However, growth of *Salmonella* species was observed in 1 culture. The isolate was characterized as *Salmonella* Agama (*S. enterica* subspecies *enterica* serotype Agama 4,12: i: 1,6). It was sensitive to ampicillin, cefotaxime, cefuroxime, ceftriaxone, imipenem, ciprofloxacin, gentamicin, trimethoprim-sulfamethoxazole, and fosfomycin but resistant to clarithromycin (MIC 96 mg/L). Five weeks after clinical resolution, further stool samples were found to be negative for any enteric pathogen.

In the light of the microbiologic evidence of *S.* Agama infection, we interviewed the patient about any consumption of meat or poultry and contact with animals. The patient reported no contact with animals during and after his trip to Gabon and said he is a vegetarian who abstains from consumption of any meat, including poultry. In Gabon, lizards are plentiful around all habitations, including the terrace of the house where the patient stayed; he reported that he ate sitting on the floor of the terrace. Lizards are also sometimes seen in food displays at street markets, including among foods that are commonly eaten uncooked (Figure).

**Figure:**
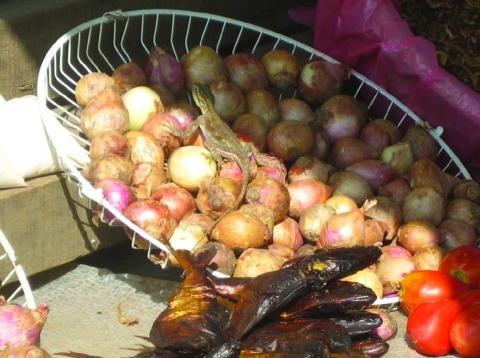
Photograph taken at a local street market in Gabon shows a lizard in a basket of onions, which are frequently eaten uncooked. *Salmonella* *enterica* subspecies *enterica* serotype Agama has been isolated from lizards in Africa.

Given microbiologic results and travel history, *S.* Agama was the most likely cause for the gastroenteritic and unspecific symptoms experienced by our patient. We may speculate about transmission of *S.* Agama by direct or indirect contact with lizards, but other routes of transmission cannot be ruled out.

Gastrointestinal and unspecific symptoms lasted 2 weeks with undulating severity and relapsed after a latent period of another 2 weeks. Although the isolate was highly resistant to clarithromycin in vitro, the patient improved clinically as symptoms disappeared. Results of stool cultures taken 5 weeks after resolution of clinical symptoms were negative. The clinical course of this patient’s illness suggests that *S.* Agama may cause self-limiting infections and asymptomatic shedding, as do other nontyphoidal Salmonella infections. The course of disease may be affected by the ingested infective dose, host factors, and virulence of *S.* Agama isolates.
